# Interactions of Influenza and SARS-CoV-2 with the Lung Endothelium: Similarities, Differences, and Implications for Therapy

**DOI:** 10.3390/v13020161

**Published:** 2021-01-22

**Authors:** Elyse Latreille, Warren L. Lee

**Affiliations:** 1 Department of Laboratory Medicine and Pathobiology, University of Toronto, Toronto, ON M5S 1A8, Canada; elyse.latreille@mail.utoronto.ca; 2 Keenan Centre for Biomedical Research, St. Michael’s Hospital, Toronto, ON M5B 1W8, Canada; 3 Interdepartmental Division of Critical Care and the Department of Medicine, University of Toronto, Toronto, ON M5B 1T8, Canada; 4 Department of Biochemistry, University of Toronto, Toronto, ON M5S 1A8, Canada

**Keywords:** SARS-CoV-2, influenza, endothelium, vascular stability, lung injury

## Abstract

Respiratory viruses such as influenza and severe acute respiratory syndrome coronavirus 2 (SARS-CoV-2) are a constant threat to public health given their ability to cause global pandemics. Infection with either virus may lead to aberrant host responses, such as excessive immune cell recruitment and activation, dysregulated inflammation, and coagulopathy. These may contribute to the development of lung edema and respiratory failure. An increasing amount of evidence suggests that lung endothelial cells play a critical role in the pathogenesis of both viruses. In this review, we discuss how infection with influenza or SARS-CoV-2 may induce endothelial dysfunction. We compare the effects of infection of these two viruses, how they may contribute to pathogenesis, and discuss the implications for potential treatment. Understanding the differences between the effects of these two viruses on lung endothelial cells will provide important insight to guide the development of therapeutics.

## 1. Introduction

**Figure 1:**
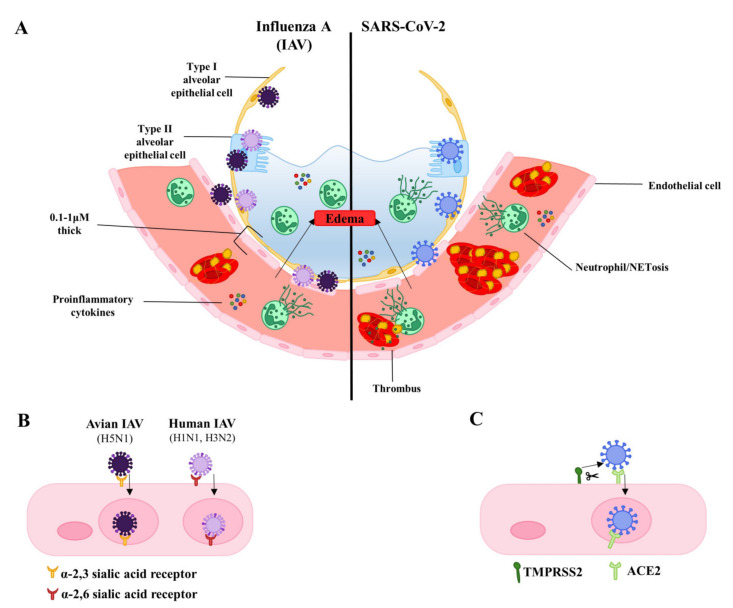
Influenza A compared to severe acute respiratory syndrome coronavirus 2 (SARS-CoV-2) infection. (**A**). On the left, infection with influenza A virus is illustrated, while SARS-CoV-2 is depicted on the right. Alveolar epithelial and endothelial cells are infected leading to proinflammatory cytokine production, recruitment of neutrophils and cell death by neutrophil extracellular traps (NETosis), activation of platelets and formation of thrombi, loss of junctional integrity and edema. (**B**). Binding of avian influenza and human influenza to their respective sialic acid linked receptors on endothelial cells. Lung endothelial infection by H3N2/H1N1 has been observed mostly in vitro and its true incidence during in vivo infections is uncertain. (**C**). Binding of SARS-CoV-2 to ACE-2 (angiotensin converting enzyme 2), its receptor, on an endothelial cell. Spike protein undergoes priming by transmembrane protease serine 2 (TMPRSS2) prior to internalization.

The maintenance of the endothelial barrier is essential for organ function. Under normal conditions, barrier integrity is tightly regulated allowing for controlled exchange of fluids, macromolecules, and cells between the circulation and tissues. However, many signaling molecules, toxins, and pathogens can induce endothelial dysfunction, causing increased permeability, proinflammatory and pro-thrombotic environments, oxidative stress, and changes in proliferation and cell death. The resulting compromise in endothelial barrier integrity is associated with several acute and chronic pathological conditions. This is particularly dramatic in the lung, where the alveolar-capillary membrane is optimized for gas exchange but vulnerable to increased permeability. Severe infections with human influenza or severe acute respiratory syndrome coronavirus 2 (SARS-CoV-2) are characterized by increased leakiness of the alveolar-capillary membrane, leading to alveolar flooding and respiratory failure. The latter syndrome is known clinically as the acute respiratory distress syndrome (ARDS) [1]. The role of the lung microvascular endothelium during respiratory infections is typically overshadowed by emphasis on the respiratory epithelium, the target cell for many respiratory pathogens [2,3,4,5]. Comparably little is known about the endothelium, even though it is an equal player in the alveolus. Dysfunction of the endothelium—whether manifesting as loss of barrier integrity or inappropriate activation—can be catastrophic [6]. In this review, we discuss how the alveolar endothelium is affected during severe infections with human influenza viruses and SARS-CoV-2, the causative agent for coronavirus disease (COVID-19) (Figure 1A, Table 1).

## 2. Virus Background

### 2.1. Influenza

Influenza viruses are segmented, single-stranded, negative sense RNA viruses belonging to the Orthomyxoviridae family. They are classified as type A, B, C, or D. For the purpose of this review, we will focus on Influenza A virus (IAV), as it commonly circulates in humans. IAV genomes are composed of 8 segments, approximately 13.5 kb altogether [7]. They can be further classified into subtypes depending on the properties of their glycoproteins, hemagglutinin (HA), and neuraminidase (NA), which protrude from the surface of the viral envelope. There are now 18 HAs and 11 NAs that have been described, and they can be found in many different combinations in human and animal host organisms. HA is responsible for binding and entry of the virus to host cells and is the target of neutralizing antibodies induced by vaccination [8]. NA cleaves and removes sialic acid from host cells and newly produced virions. As such, it is required for release of newly produced virions and spread to other host cells [9]. One of the two classes of currently used antivirals acts by inhibiting this protein, thereby limiting the spread of the virus to other host cells [10]. H1N1 and H3N2 are the most commonly circulating influenza A viruses in humans, causing seasonal outbreaks. In addition, H1N1 can cause pandemics such as the Spanish flu of 1918 that infected an estimated 500 million people, killing 50 million [11]. Highly pathogenic avian influenza (HPAI) such as H5N1, among others, has been causing an increasing amount of sporadic infections in humans. While the true case fatality rate of viruses such as H5N1 is unknown, they undoubtedly have a higher fatality rate than seasonal and pandemic influenza. Indeed, estimates of the case fatality rate of H5N1 range from 14% to as high as 60%, making it a serious pandemic threat. However, H5N1 is not yet capable of efficient human to human transmission, limiting the spread of the virus [12,13]. In addition to the high mutation rate of influenza, it can also undergo genome reassortment as it is a segmented virus. For example, in a host infected with two different subtypes of influenza A, genetic segments of the two viruses may be mixed, giving rise to a novel virus that may have pandemic potential [8]. As such, the identification of novel therapies for the treatment of severe influenza is urgently needed to be prepared for the possible emergence of a novel pandemic strain.

### 2.2. SARS-CoV-2

SARS-CoV-2, the causative agent of COVID-19, is a single stranded, positive sense RNA virus belonging to the Coronaviridae family, of the beta genera. While four genera exist, the alpha (hCOV-229E and hCOV-NL63) and beta coronaviruses (hCoV-OC43 and hCoV-HKU1) circulate and cause regular seasonal outbreaks of the common cold in humans. Betacoronaviruses such as SARS-CoV and Middle Eastern respiratory syndrome coronavirus (MERS-CoV) have also caused more serious epidemic outbreaks in the past. Coronaviruses have the longest genomes among RNA viruses, at 26–32 kb. They also possess an envelope, with spike (S), envelope (E), and membrane (M) proteins. Spike protein protrudes from the surface of the virion and is responsible for receptor binding and entry [14,15]. As such, vaccines against SARS-CoV-2 are designed to elicit spike-targeting antibodies.

Bats are the natural animal hosts of pathogenic coronaviruses, and spillover to humans is thought to occur via intermediate hosts, as occurred with the civet and SARS-CoV and with dromedary camels and MERS-CoV [14]. SARS-CoV first emerged in November 2002 in China and rapidly spread to 26 countries before it ended in July of 2003 and has not caused any human cases since. During this time, SARS-CoV caused 8098 cases and 774 deaths, giving it a mortality rate of 9.6% [16]. In contrast, MERS-CoV first emerged in April 2012 in Saudi Arabia, continues to cause sporadic outbreaks, and has the potential to spread globally. Between April 2012 and December 2019, it caused 2499 confirmed cases with 858 deaths in 27 countries. This puts its mortality rate around 34.3%. While the true mortality rate of MERS-CoV is uncertain, it has been estimated to be around 29.8% in cases from Saudi Arabia between 2014 and 2016, with a 45.2% mortality rate in patients over 60 [17]. While SARS-CoV, MERS-CoV, and SARS-CoV-2 can cause similar severe illness, SARS-CoV-2 is thought to be more transmissible, but less lethal [15]. Indeed, SARS-CoV-2 has now infected over 82.1 million worldwide and caused 1.79 million deaths as of December 2020. Its mortality rate is currently estimated to be around 2% [18].

## 3. Infection of the Lung Endothelium by the Virus: Frequency and Potential Relevance to Pathology

### 3.1. Cell Death and Endothelial Permeability

A key issue for both viruses is the incidence and pathological significance of lung endothelial infection. Both α-2,6 and α-2,3-linked sialic acid residues, the receptors for human and avian influenza respectively, are expressed by human lung endothelial cells (Figure 1B) [19,20,21]. As such, both commonly circulating influenza and highly pathogenic avian influenza viruses can infect and replicate in human endothelial cells. The spread of influenza infection from the upper to lower respiratory tract is thought to contribute to the development of severe disease [22]. In human lungs, α-2,3-linked sialic acid residues are predominantly expressed in the lower respiratory tract. This may explain why H5N1 often infects the lower respiratory tract causing severe pneumonia and poor outcomes [23]. It has been proposed that enhanced endothelial tropism of H5N1 contributes to disease severity and poor outcomes in poultry and mice [24,25]. Despite the expression of both α-2,6 and α-2,3-linked sialic acid residues by lung endothelial cells, H5N1 has been shown to be more efficient at binding and replicating in human pulmonary microvascular endothelial cells than H1N1 viruses [26].

Direct infection may contribute to endothelial dysfunction. Recently, Tundup et al. created H5N1 viruses with restricted cell tropism and demonstrated the contribution of endothelial cell infection to edema and proinflammatory cytokine production during severe H5N1 infection. Infection of mice with an H5N1 strain that was unable to replicate in endothelial cells led to reduced vascular leakage and proinflammatory cytokine levels in the lungs. The results from this study also suggest that infection of leukocytes plays a non-negligible role in cytokine production. Infection with leukocyte replication-deficient virus caused an intermediate phenotype between the endothelial replication-deficient and control virus for cytokine levels, vascular leakage, and lung damage. Interestingly, this intermediate phenotype was not accompanied by improved survival, as only animals infected with the endothelial-replication restricted virus displayed improved survival. Importantly, animals infected with any of the viruses displayed similar viral titer in the lungs, suggesting that the host response (rather than viral clearance per se) plays an important role in the pathogenesis of the virus [25].

While lung endothelial infection by avian influenza viruses such as H5N1 plays an important role in pathogenesis, there is less direct evidence of human influenza viruses such as H1N1 and H3N2 infecting lung endothelial cells. H3N2 infection of human lung endothelial cells has been shown to induce endothelial apoptosis and increase endothelial permeability in vitro, which can be reduced by the inhibition of caspases [27]. The mechanisms by which influenza induces endothelial apoptosis remain unclear. Extensive work in epithelial cells has revealed the ability of several viral proteins such as influenza NP, NS1, and to induce apoptosis by different mechanisms [28,29,30]; whether similar mechanisms occur in endothelial cells has yet to investigated. In addition, there is pre-clinical evidence of in vivo endothelial infection by H3N2. Staining for influenza virus nucleoprotein has been shown to colocalize with endothelial membrane protein PECAM-1 in the lungs of infected mice in a dose-dependent manner [31]. While others have also shown that A(H1N1)pdm09 can infect endothelial cells, the levels of influenza nucleoprotein detected in endothelial cells were low compared to mice infected with avian H5N1 [32]. As such, studies investigating direct endothelial infection by non-avian influenza are limited. The frequency and consequences of direct endothelial infection by human influenza viruses are unclear. Our group has previously reported that exposure to very low inocula of influenza A was sufficient to sensitize or prime the lung microvascular endothelium to become more permeable when subsequently challenged with *Staphylococcus aureus*, even when the pathogens were given days apart. Sequential exposure to influenza and *S. aureus* induced a significant increase in endothelial leakage which was recapitulated in a murine model; the mechanism involved TNFR1-mediated lung endothelial apoptosis [33]. As a major complication of influenza infection is bacterial superinfection in the lungs (e.g., with *S. aureus* or *S. pneumoniae*), these data provide a potential explanation for how infection with the influenza virus may predispose patients towards developing ARDS upon challenge with a bacterial pathogen [34].

Unlike the uncertainty with many strains of influenza virus, it is very clear that endothelial cells inside and outside of the lung can be infected by SARS-CoV-2 [35,36,37,38,39]. The S1 subunit of the SARS-CoV-2 spike protein contains the receptor binding domain and is responsible for virus binding to angiotensin-converting enzyme 2 (ACE2) expressed on the host cell surface. Receptor binding alone is not sufficient for viral entry, as SARS-CoV-2 spike protein must be primed by host proteases such as surface-expressed transmembrane protease serine 2 (TMPRSS2). Cleavage of the spike protein at the S1/S2 and S2′ sites by TMPRSS2 allows for release of S1 and induces structural changes in the S2 subunit. This leads to S2-driven viral and cellular membrane fusion and subsequent viral entry (Figure 1C) [40]. TMPRSS2 has been shown to be essential, as its inhibition blocks SARS-CoV-2 entry [41]. Endothelial cells have been shown to express angiotensin converting enzyme 2 (ACE2), the receptor for SARS-CoV and SARS-COV-2, in human tissue biopsy samples from various organs [42]. TMPRSS2 has also been shown to be expressed by endothelial cells in culture and in blood vessels of immunostained human respiratory tissues [41,43,44,45]. Transmission electron microscopy of the endothelium from lung tissues of COVID-19 patients shows the presence of intracellular SARS-COV-2, further supporting the ability of the virus to infect endothelial cells [46]. As is the case with influenza, infection of endothelial cells by SARS-COV-2 is associated with endothelial apoptosis, as evidenced by staining of tissues from patients deceased of COVID-19-associated respiratory failure [47]. While observational findings in humans provide evidence of SARS-CoV-2 infection and apoptosis of the endothelium, there is currently a lack of direct in vitro evidence and the mechanisms of virus-induced endothelial apoptosis remain to be investigated. However, work in epithelial cells suggests some possibilities. Coronaviruses encode many accessory proteins whose functions remain unclear. It was recently shown that the SARS-CoV-2 ORF3a can induce apoptosis when transfected in epithelial cell lines. Interestingly, SARS-CoV ORF3a (i.e., from the virus causing SARS) expression induced higher levels of caspase-3 activation than SARS-Cov-2 ORF3 expression, suggesting weaker pro-apoptotic activity of SARS-CoV-2 ORF3a [48]. ORF3a may induce apoptosis via the extrinsic pathway given that expression of SARS-CoV-2 ORF3a leads to cleavage and activation of caspase-8. Other studies have found that the induction of apoptosis by SARS-CoV-2 infection requires caspase 8, as caspase 8 inhibitors greatly reduce BID cleavage, caspase-3 activation, and PARP-1 cleavage upon infection with SARS-CoV-2 [49]. Other SARS-CoV proteins such as ORF7a and the structural membrane protein have been found to induce apoptosis, but this has yet to be investigated for SARS-CoV-2 [50,51].

### 3.2. Inflammatory Cell Death

In addition to apoptosis, cells may die via pyroptosis, a caspase-1-dependent inflammatory cell death pathway. The NLRP3 inflammasome can be activated by viral infection, leading to caspase 1 activation, pore formation in the plasma membrane, and subsequent loss of ionic gradients leading to cell swelling and rupture. This is accompanied by release of proinflammatory content, such as IL-1β, and cell death [52]. In the context of influenza infections, the role of the NLRP3 inflammasome and pyroptosis in relation to disease is controversial. It has been suggested that NLRP3 activation may contribute to endothelial barrier dysfunction and pathogenesis due to the increased release of IL-1β and the activation of NF-κB. This results in enhanced cytokine, chemokine, and adhesion molecule production. In support of this hypothesis, influenza A PB1-F2 protein has been shown to activate the NLRP3 inflammasome. Mice infected with PB1-F2 deficient virus exhibited reduced levels of proinflammatory cytokine IL-1β. Exposure to PB1-F2 peptide from pathogenic influenza increased IL-1β secretion compared to PB1-F2 from seasonal influenza. This implicates the activation of the NLRP3 inflammasome in influenza A virus-associated pathogenesis [53]. However, knockout of NLRP3 or caspase-1 in mice has been shown to increase morbidity and mortality during influenza infection. It has been suggested that NLRP3 activation may play an essential role during IAV infection in the detection of viral RNA and the recruitment of leukocytes to the infected lung [54,55].

The activation of the NLRP3 inflammasome during coronavirus infection is thought to contribute to pathogenesis. At least three SARS-CoV viral proteins have been shown to activate the NLRP3 inflammasome by acting as ion channels, priming NLRP3 activation via NF-κB, and by direct interaction with NLRP3 [56,57]. While the ability of SARS-CoV-2 proteins to activate the NLRP3 inflammasome and induce pyroptosis remain to be investigated, it is likely that it has similar mechanisms as the two viruses share 79.6% sequence identity [58]. This is supported by the recent finding that SARS-CoV-2 induces caspase-1 activation, IL-1β secretion, and pyroptosis in cultured human primary monocytes. Monocytes isolated from COVID-19 patients have also been found to have increased caspase-1 activation and cell death compared to those from healthy donors, suggesting inflammasome activation and pyroptosis may be involved in COVID-19 [59]. However, whether SARS-CoV-2 induces pyroptosis in lung endothelial cells, and if this contributes to pathogenesis, has yet to be investigated. A recent study suggests that endothelial pyroptosis may play a more important role in the pathogenesis of SARS-CoV-2 than A(H1N1)pdm09, as caspase 1 was found to be elevated in lung endothelial cells from tissue samples of COVID-19 patients compared to H1N1 patients and control samples. It has been suggested that enhanced endothelial pyroptotic death during SARS-CoV-2 infection may contribute to the development of systemic thrombosis observed in COVID-19 patients [60].

While it is apparent that lung endothelial cells can be infected with SARS-CoV-2, as with influenza, the impact of frank endothelial cell infection during COVID-19 remains unclear. While infection of the alveolar capillary endothelium could contribute to pulmonary edema and venous thromboembolism, other mechanisms such as cytokine-induced endothelial activation or cell death are likely to be involved. In addition, it is currently thought that lung epithelial cell death plays a major role in the induction of vascular leak within the alveoli [61].

## 4. Indirect Endothelial Damage and Dysfunction—The Role of Cytokines and Inflammation

Infection with IAV or SARS-CoV-2 is associated with the production of proinflammatory cytokines by host cells. Elevated inflammatory cytokines may be involved in the induction of endothelial leak and contribute to pathogenesis.

One study compared lung RNA from lethal versus non-lethal infections with influenza virus in mice in an attempt to identify host factors that correlated with fatal infections. They observed that fatal infections exhibited a distinctive transcriptional signature that was accounted for in large part by neutrophil influx into the lungs. The authors reported what they called a “chemokine-driven feed-forward circuit involving proinflammatory neutrophils” in the setting of lethal influenza infection. Intriguingly, partial depletion of neutrophils using antibodies improved the survival of infected animals without affecting viral titer in the lung. These data strongly implicate an aberrant or excessive host response in determining outcome at least in these pre-clinical models of severe influenza [62].

Accordingly, the increased pathogenesis of avian influenza viruses such as H5N1 may be due to the excessive cytokine response. H5N1 infection is associated with elevated levels of proinflammatory cytokines and chemokines such as MCP-1, IL-8, IL-10, and IL-6, among others, in the peripheral blood of H5N1 patients compared to H1N1 and H3N2 infected patients. Levels were found to be particularly elevated in patients that died, but viral load was also higher [63]. It was previously thought that leukocytes and epithelial cells were predominantly responsible for the excessive production of cytokines during influenza infection. However, evidence now supports the hypothesis that endothelial cells are important for the initiation and amplification of the cytokine storm. The endogenous lipid, sphingosine-1-phosphate (S1P), has been implicated in the regulation of endothelial-directed cytokine production during influenza infection. Specifically, Teijaro et al. demonstrated that administration of CYM-5442, an S1P_1_ receptor-selective agonist, significantly improved survival of influenza-infected mice. The drug exerted its effects by acting on lung endothelial S1P_1_, reducing endothelial cytokine and chemokine production and leukocyte recruitment. Although S1P was initially described as a mediator of lung vascular stability [64], the effect of S1P_1_ agonism to improve survival of infected mice appeared to be independent of an effect on lung endothelial barrier function. Importantly, even when leukocyte recruitment was restored, this was not sufficient to restore cytokine and chemokine levels in the lungs of infected mice. This suggests that endothelial cells, not epithelial cells and leukocytes, play a dominant role in the early production of cytokines and chemokines as well as leukocyte recruitment during influenza infection [65].

Cytokines likely also play a role in the pathogenesis of COVID-19. In SARS-CoV-2-infected patients from Wuhan, levels of IL-6 were elevated in samples from deceased compared to discharged patients, suggesting that enhanced production of IL-6 may contribute to the severity of disease [66]. In another study, IL-6 was found to be nearly 10 times higher in critically ill compared to severe patients, suggesting IL-6 may be an indicator of poor outcomes [67]. IL-6 may exacerbate inflammation and cause further leukocyte recruitment, as it has been shown to induce the expression of other proinflammatory cytokines, chemokines, and adhesion molecules in endothelial cells [68,69]. IL-6 has both a membrane bound receptor (IL-6R) and soluble receptor released into the circulation (sIL-6R) and can bind to both receptors with similar affinity. Downstream signaling through the membrane bound receptor is associated with anti-inflammatory and regenerative functions of IL-6, while signaling through the soluble form of the receptor (IL-6 trans-signaling) is associated with the proinflammatory functions of IL-6 [70]. Blockade of IL6 trans-signaling has been shown to reduce inflammation in multiple animal models, including endotoxin-induced shock, intestinal inflammation, atherosclerosis, and cancer [71,72,73,74,75,76]. Elevated IL-6 may also contribute to SARS-CoV-2-related pathogenesis by inducing endothelial barrier dysfunction, as treatment with IL-6 has been shown to induce permeability changes in endothelial cell monolayers. While the specific mechanisms of IL-6-induced permeability remain controversial, it may induce permeability by altering the endothelial cytoskeleton or adherens and tight junctions. Endothelial cells exposed to IL-6 display reduced junctional localisation of the tight junction component Zonula occludens-1 (ZO-1), increased phosphorylation of vascular endothelial (VE)-cadherin (the major component of adherens junction), loss of VE-cadherin at intercellular junctions, disorganised cytosolic actin, and increased cell contraction [77,78]. Additional studies are required to further elucidate which cell types are the major contributors to the excessive production of proinflammatory cytokines during SARS-CoV-2 infection and their contribution to pathogenesis. Interestingly, infection of macaques with 1918 H1N1 was found to greatly increase IL-6 levels compared to macaques infected with conventional H1N1, suggesting IL-6 may also play an important role in the pathogenesis of severe influenza [79].

Of note, the elevated cytokines seen in some patients with COVID-19 has led to speculation that tissue damage is attributable to a “cytokine storm”. While cytokines undoubtedly contribute to pathology in this infection, the levels of cytokines in COVID-19 are not more elevated than in other critically ill patients [80,81]. Furthermore, not all patients display elevations in cytokines prior to evidence of organ dysfunction [82].

## 5. Consequences of Endothelial Disruption—Loss of Junctional Integrity

**Figure 2:**
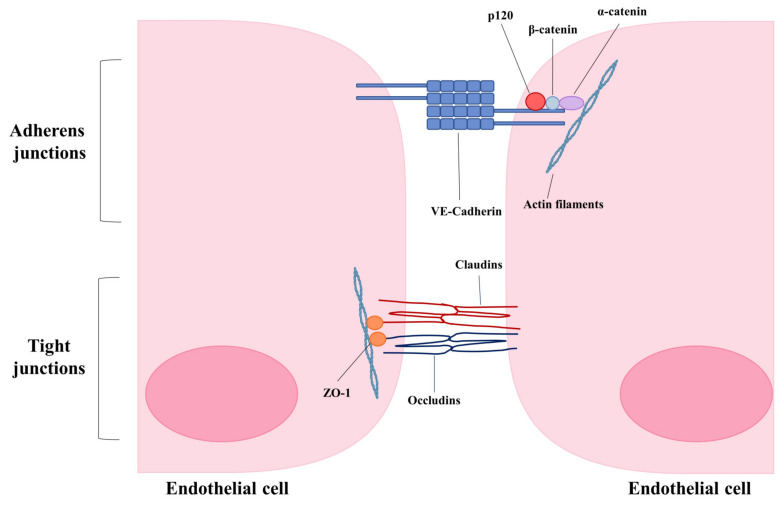
Schematic of major components of endothelial adherens and tight junctions.

As alluded to earlier, endothelial cell-cell junctions play a critical role in the maintenance of vascular integrity by controlling permeability (Figure 2). Remodeling of endothelial cell adherens and tight junctions is a major contributor to increased endothelial permeability and altered vascular integrity.

In the past there has been concern that tightening of the endothelial barrier might be detrimental during ARDS by preventing leukocyte transmigration to the site of infection and impairing the host immune response. Accumulated data now suggest that leukocyte emigration and endothelial permeability can be manipulated separately, implying that vascular leakage may be attenuated without impairing clearance of the pathogen [83]. This has now been reported by a number of groups using different pre-clinical models of infection and inflammation [84,85]. It is therefore critical to understand the fundamental mechanisms of vascular permeability so that additional therapeutic strategies may be developed.

VE-cadherin is the major protein of endothelial adherens junctions. It connects neighboring endothelial cells in a calcium-dependent manner by its extracellular domain and provides anchorage to the cytoskeleton by binding with catenin proteins via its intracellular domain [1,86,87,88]. Binding of p120 to the intracellular domain of VE-cadherin is essential for junctional integrity as it selectively inhibits clathrin-mediated endocytosis of VE-Cadherin [86]. Depletion of p120 has been shown to reduce transendothelial resistance, indicating that the internalization of VE-cadherin from the plasma membrane destabilizes adherens junctions and increases vascular permeability [89]. Many inflammatory mediators such as vascular endothelial growth factor (VEGF) can induce the phosphorylation and endocytosis of VE-cadherin through Src kinase and Rac GTPase, thereby disrupting endothelial barrier function [90]. Proinflammatory cytokines such as IL-1β have also been shown to rapidly induce permeability via VE-Cadherin internalization in cultured endothelial cells [91]. Given that infection by viruses such as influenza and SARS-CoV-2 cause loss of vascular integrity leading to pulmonary edema, enhancing endothelial barrier integrity may offer a potential therapeutic strategy. Strengthening endothelial barrier integrity has been shown to improve survival in many animal models characterized by lung edema, such as polymicrobial sepsis, endotoxin exposure, and infection with highly pathogenic avian influenza (H5N1) [92]. For example, the administration of an active fragment of the neuronal guidance protein Slit (Slit2N) restored the association of p120 catenin and VE-cadherin, localization of VE-cadherin to the cell surface and reduced permeability in vitro. In mouse models of sepsis and H5N1 infection, administration of Slit2N to mice significantly improved survival and reduced endothelial hyperpermeability [92].

Tie2 is an angiopoietin (ANG) receptor present on the cell surface of endothelial cells. ANG1 is a Tie2 agonist, and its binding to Tie2 induces signaling associated with cell survival, vessel stability, and endothelial barrier function. ANG2 attenuates ANG1-Tie2 signaling and can act as a Tie2 antagonist during inflammation, leading to destabilized endothelial monolayers and promoting vascular leakage [93,94]. In one study, treatment of mice with Vasculotide, a novel Tie2 agonist, significantly improved survival of H1N1-, H3N2-, and A(H1N1)pdm09-infected mice without affecting viral titer. This compound reduced endothelial leak induced by thrombin in vitro and influenza induced lung endothelial cell death both in vitro and in vivo [84]. While the effect of Vasculotide on VE-cadherin localization was not investigated in the context of this study, Ang1, the endogenous ligand of Tie2, has been shown to increase VE-cadherin localization to adherens junctions and prevent VEGF-induced permeability [95]. In addition, others have shown that Vasculotide enhances VE-cadherin junctional localization and prevents intercellular gap formation in endotoxin-treated endothelial cells and stabilizes VE-cadherin in a model of renal ischemia-reperfusion injury in mice [96,97].

Endothelial tight junctions are composed of claudins, occludins, and junctional adhesion molecules. Alterations of tight junctions during infection may also contribute to endothelial leak and pulmonary edema. For example, treatment of lung endothelial cells with replication-deficient influenza virus has been shown to induce endothelial permeability, independent of cell death. Infection with replication-deficient virus was found to induce the loss of claudin-5 protein levels in vitro via degradation by matrix metalloproteinases (MMPs). Loss of claudin-5 was required for the observed increase of permeability, as over-expression of claudin-5 or treatment with formoterol, a compound that induces claudin-5 expression, was sufficient to prevent replication-deficient influenza induced endothelial leak. In addition, treatment of influenza-infected mice with formoterol reduced pulmonary edema [98].

SARS-CoV-2 induced loss of endothelial barrier integrity may be due to its ability to interfere with endothelial tight or adherens junctions. A recent study investigated the effects of plasma from healthy donors compared to moderate or severe COVID-19 cases on endothelial permeability. In vitro treatment of endothelial cell monolayers with plasma from COVID-19 patients increased endothelial permeability, caused the loss of junctional VE-cadherin and cortical actin, and induced actin stress fiber and inter-cellular gap formation within hours of exposure. Additionally, exposure of human lung tissue from donors dying of non-COVID-related causes to plasma from severe COVID-19 cases significantly reduced levels of the tight junction protein occludin. Given that plasma used in this study was negative for the SARS-CoV-2 virus, these findings support the notion that pathology in severe COVID-19 is caused by the aberrant host response rather than by direct injury by viral infection [99].

A study investigating the role of SARS-CoV-2-spike protein on the blood brain barrier found that spike could induce permeability of endothelial monolayers and leakage in a 3D blood brain barrier model. ZO-1 was found to have reduced localization to tight junctions in spike protein-exposed vessels compared to untreated vessels, potentially explaining the loss of integrity of endothelial tight junctions. SARS-CoV-2 spike protein also induced the expression of adhesion molecules such as intercellular adhesion molecule 1 (ICAM-1) and vascular cell adhesion molecule 1 (VCAM-1), as well as proinflammatory cytokines and chemokines such as IL-1β, IL-6, CCL5, and CXCL10, suggesting that spike protein is inducing a proinflammatory phenotype in endothelial cells [100]. Further work will be required to tease apart the contribution of the effects on junctional proteins and inflammatory protein expression on spike-induced endothelial permeability and their contribution to pathogenesis.

## 6. Endothelial-Platelet Interactions and Coagulopathy

### 6.1. Influenza

A major function of the resting endothelium is the prevention of unnecessary thrombosis. Platelet aggregation and pro-coagulant effects can be induced by infected, activated endothelial cells. In vitro studies have demonstrated that infection of endothelial monolayers with influenza virus increases tissue factor (TF) expression. TF is an important pro-coagulant protein involved in the generation of thrombin, the main effector protease of the coagulation cascade. Endothelial influenza infection reduced clotting time by 55% as quickly as three hours post infection [101]. Similarly, H1N1 infection in mice increased TF levels in the lungs and increased thrombin-antithrombin complexes in the bronchoalveolar lavage, indicating activation of coagulation [102]. H1N1 infection also increases thrombin production, fibrin deposition, and fibrinolysis in mice, indicating activation of coagulation and induction of a pro-thrombotic state [103]. H1N1 can also form immune complexes with IgG to directly activate platelets via FcγRIIA, or independently of immune complexes via thrombin [104]. Increased platelet activation and adhesion have been implicated in the pathogenesis of severe influenza in murine models. Large platelet aggregates have been reported in the lungs of influenza-infected mice. Knockout of GPIIIa, an essential factor for platelet aggregation, reduced lung injury and improved survival of infected mice, suggesting platelet aggregation contributes to the pathogenesis of influenza. In addition, antagonism of PAR4, an important receptor for platelet activation, also improved outcomes of infected mice, while its stimulation increased inflammation and worsened outcomes [105]. The involvement of endothelial cells was not investigated in this study. However, it has been reported that infection of endothelial cells with influenza induces platelet adhesion both to cultured lung endothelial cells and in lungs of infected mice. In this study, platelet-endothelial interactions were shown to be involved in pathogenesis, as treatment of mice with compounds known to impair platelet function and reduce platelet-endothelial interactions significantly improved survival of infected mice [31]. In humans, the extent to which abnormalities in the coagulation system contribute to pathogenesis is unclear. Analyses of a small number of severe or fatal cases of the 2009 H1N1 pandemic flu revealed the presence of pulmonary thrombi and pulmonary emboli with or without deep vein thrombosis [106,107]. However, the importance of coagulation abnormalities is controversial as other, larger retrospective studies from patients with influenza A or B found only a small percentage of patients had thrombotic events. Furthermore, influenza infection was not associated with increased risk of pulmonary embolism [108,109].

### 6.2. SARS-CoV-2

In contrast to the uncertainty around influenza, there have been numerous reports of derangements of coagulation in COVID-19 patients. Comparison of lungs from patients deceased of COVID-19 or of influenza-induced ARDS indicate increased thrombi in pulmonary arteries and fibrin thrombi in the alveolar capillaries of both groups compared to control samples. However, alveolar capillary microthrombi were 9 times more prevalent in the COVID-19 group than in the influenza group [46].

Similarly, 57% of COVID-19 and 58% of SARS cases had pulmonary microthrombi compared to only 24% in H1N1 cases. Additionally, 15% of COVID-19 and 28% of SARS autopsy cases, but only 6% of H1N1 cases, were found to have thrombosis in medium to large pulmonary vessels [110]. COVID-19 cases have also been reported to have a much higher occurrence of immunothrombosis (activation and interaction of platelets, neutrophils, and NETs regulated by coagulation and inflammatory mediators), with 40.8% of vessels affected by immunothrombotic occlusions in COVID-19 compared to only 9.4% in influenza [111].

In one study of over 3000 hospitalized patients with COVID-19, 16% developed a thrombotic event, of which 2/3 were arterial in location. In the subset of patients in the intensive care unit, almost 30% had a thrombotic event. These thromboses occurred despite prophylactic therapy and seem significantly higher than observed in patients with influenza [112]. Similarly, comparison of COVID-19 ARDS patients to non-COVID-19 ARDS patients revealed significantly more thrombotic complications in patients with COVID-19, including the increased occurrence of pulmonary emboli. Patients with severe COVID-19 have also been reported to have lowered platelet counts, abnormal coagulation parameters such as increased prothrombin time and elevated D-dimers, and increased incidence of disseminated intravascular coagulation when compared to the non-severe group [113]. Lowered platelet counts during SARS-CoV-2 infection are thought to be caused by either reduced platelet production, increased platelet destruction, or increased platelet consumption due to the formation of aggregates and microthrombi [114]. Both platelet and endothelial cell activation markers have been found to be elevated in COVID-19 intensive care unit (ICU) patients compared to non-ICU patients. Non-ICU patients with COVID-19 were found to have elevated von Willebrand factor (vWF) levels and activity as well as D-dimer and thrombin-antithrombin levels above the normal range, which were further increased in ICU patients. Interestingly, mortality correlated with elevated vWF and soluble thrombomodulin, a marker of endothelial injury. These findings support the hypothesis that coagulopathy may be induced by endothelial injury and contributes to the severity of COVID-19 [115,116].

Interestingly, the SARS-CoV-2 virus itself has recently been shown to directly activate platelets, which express ACE2 and TMPRSS2. SARS-CoV-2 virions have been shown to directly bind to platelets via the spike protein and activate them through MAPK signaling. Importantly, binding of SARS-CoV-2 or spike protein alone was able to induce platelet aggregation in response to factors such as thrombin and collagen. SARS-CoV-2 and spike protein were also able to induce the release of coagulation factors and inflammatory cytokines such as TNF-α, IL-8, IL-1β, and PF4, expression of adhesion molecule P-selectin, and formation of platelet-neutrophil and platelet monocyte aggregates. Spike protein also activated platelets in vivo, as thrombus formation was evident in mice transfused with platelets from humanized ACE2 transgenic mice [117].

Enhanced platelet adhesion and activation during both influenza and SARS-CoV-2 infection may also amplify the inflammatory response and result in further endothelial activation, vascular leak and disseminated intravascular coagulation [118]. Activated platelets can release inflammatory cytokines and chemokines such as CD40L, IL-1β, CCL5, CXCL4, CXCL7, and TGF-β, among others. This can in turn induce endothelial expression of cell adhesion molecules such as ICAM-1, VCAM-1, E-Selectin, and P-Selectin in addition to proinflammatory cytokines and chemokines such as IL-6, IL-8 and MCP-1 (CCL2) [119,120,121,122].

## 7. Neutrophils and Neutrophil Extracellular Traps

### 7.1. Influenza

Increased endothelial expression of adhesion molecules such as ICAM-1, VCAM-1, P-Selectin, and E-Selectin allows for the recruitment of leukocytes to the site of infection. While this is essential for the innate immune response to infection to limit viral replication, it can result in lung damage if excessive. For instance, enhanced infiltration of macrophages and neutrophils has been reported in mice infected with highly pathogenic strains of influenza such as 1918 H1N1 and H5N1, compared to low pathogenic strains, along with higher levels of proinflammatory cytokines and chemokines such as MCP-1, MIP-1a, IL-1a, IFN-y, and IL-6 [123]. Enhanced neutrophil recruitment is specifically thought to play an important role in the development of acute respiratory distress syndrome in severe influenza. In a study of 441 patients with A(H1N1)pdm09, patients with pneumonia had elevated absolute neutrophil counts compared to those without pneumonia [124]. As mentioned earlier, in murine models of fatal influenza infection, mortality correlated with neutrophil influx to the lungs. Importantly, this study found neutrophil extracellular traps (NETs) in areas of tissue injury in the lungs [125].

NETs are extracellular structures, released by activated neutrophils, that are primarily composed of proteins and decondensed chromatin [126]. While NETs are thought to play a role in host defense, they are also associated with a number of deleterious effects such as tissue damage, hypercoagulability, and thrombosis [127]. They are thought to directly cause endothelial and epithelial cell death, promote thrombosis by acting as a scaffold and activating platelets, recruit pro-coagulation factors, bind vWF and fibrin to recruit platelets, and modulate immune cells to enhance production of inflammatory cytokines [126,128]. In animal models of influenza, NETs have been found in pulmonary lesions and are thought to contribute to lung damage [125]. Elevated NETs have also been suggested to be a predictive marker for poor prognosis in influenza patients, as elevated NETs correlate with disease severity in H7N9- and H1N1-infected patients. Interestingly, NETs from plasma of patients with severe H7N9 and H1N1 were found to induce permeability in cultured epithelial cells, indicating that elevated NETs may contribute to edema and lung injury during influenza infection [129].

### 7.2. SARS-CoV-2

Recent findings also suggest a role for neutrophils and NETs in severe COVID-19. Neutrophil counts have been found to be elevated in severe COVID-19 cases and an increased neutrophil to lymphocyte ratio has been shown to be a predictor of severe illness [130,131]. Many studies have now reported neutrophilia, increased neutrophil infiltration in the lungs, and increased NETs in plasma and lungs of COVID-19 patients [127,131,132,133,134]. Neutrophils have also been found to be further increased in severe COVID-19 ARDS patients compared to less severe cases. While no differences were found in neutrophil recruitment and activation when compared to influenza pneumonia cases, NETosis was elevated in severe COVID-19 compared to influenza pneumonia. Additionally, vWF (a marker of endothelial injury and NETosis) was found to be increased in COVID-19 lungs compared to the normal reference range. Finally, the authors found that monocytes may also play an important role in neutrophil recruitment to the lungs during severe COVID-19, as they observed increased neutrophil-attracting chemokine production by monocytes. The authors proposed that neutrophils may play a more important role in the pathology of COVID-19, through NETosis and immunothrombosis, compared to influenza-virus induced pneumonia [111]. While the mechanisms of induction of release of NETs upon SARS-CoV-2 infection are unknown, it has been shown that infectious, but not inactivated, SARS-CoV-2 can promote NET release in an ACE2/TMPRSS2-dependent manner [134]. Elevated cytokines and chemokines in COVID-19 patients may also be responsible for the induction of NETosis.

The formation of NETs may contribute to the development of respiratory failure and severe disease by inducing endothelial damage, inflammation, or the development of coagulopathy and thrombosis. In a study of samples from patients deceased of COVID-19, neutrophilic plugs composed of NETs or with NETs and platelets, were discovered in many organs including the lungs, heart, kidneys, liver, spleen and brain [135]. Concurrently, a recent prospective cohort study of 33 COVID-19 patients and 17 age-matched controls found elevated plasma NETs in both severe and non-severe COVID-19 patients, with highest levels in non-survivors. Higher plasma NETs were found to be associated with increased respiratory failure. The authors of this study also found evidence supporting the hypothesis that NETs may be involved in thrombosis observed in COVID-19 ARDS, as neutrophils undergoing NETosis colocalized with platelets in the lungs. Elevated platelet-neutrophil aggregates and levels of NETosis-inducing factors such as IL-6, IL-8, PF4, and RANTES were also reported [127]. Formation of NETs may also contribute to disease via vessel occlusion. In autopsy-derived lung tissue from a patient with COVID-19, pulmonary vessels were found to be occluded by aggregated NETs. In addition, vessel walls near intravascular aggregated neutrophil and NETs containing clots displayed evidence of endothelial cell damage [132]. The authors suggest that SARS-CoV-2-induced endothelial damage triggers neutrophil recruitment and NET formation, however the converse is also possible (e.g., endothelial damage is a result of excessive NET formation). Regardless, NETs appear to be detrimental and have been proposed as a potential therapeutic target for treatment of COVID-19 [136].

## 8. Implications for Therapy

### 8.1. Role of Antiviral Drugs

Current treatment options for both influenza and SARS-CoV-2 remain limited. For influenza, antivirals such as neuraminidase inhibitors (Oseltamivir) and a cap-dependent endonuclease inhibitor (Baloxavir marboxil) are currently used for treatment [137,138,139]. Both drugs act by directly inhibiting viral proteins. As such, these therapies exert a selective pressure on the virus, which in combination with the high mutation rate of influenza leads to rapid emergence of antiviral resistant strains. For example, the M2 ion channel inhibitors such as adamantanes are no longer clinically useful for the treatment of influenza due to almost universal resistance [140]. Furthermore, it is recommended that antiviral therapy be administered within 48 h of infection for maximum benefit. As such, identifying therapeutics that improve the outcome of severe influenza infection by modulating the excessive, damaging host response may be a useful adjunctive approach.

Treatment options for SARS-CoV-2 are also extremely limited. The most promising antiviral for SARS-CoV-2 is Remdesivir, a nucleoside analogue that interferes with the RNA-dependent RNA polymerase of the virus by acting as a delayed chain terminator. It has been shown to reduce viral titer in vitro [141,142,143,144]. In pre-clinical studies, Remdesivir was shown to reduce viral burden, morbidity, and lung pathology of SARS-CoV infected mice when administered early after infection or prophylactically [143]. It has since been shown to be beneficial when administered early after infection of rhesus macaques with MERS-CoV or SARS-CoV-2, as demonstrated by reduced viral burden in the lungs or bronchoalveolar lavage, reduced signs of respiratory disease and reduced lung damage [145,146]. However, multiple groups have reported that Remdesivir provides no benefit in hospitalized COVID-19 patients. For example, recent trials have indicated that treatment with Remdesivir does not reduce mortality, initiation of ventilation or hospitalization [147]. It has also been reported that a 10-day course of Remdesivir has no effect on clinical status, while a 5-day course showed improved clinical status that was of debatable clinical importance [148]. Another study found that while overall, Remdesivir did not reduce time to clinical improvement, patients who received Remdesivir within 10 days of symptom onset had a shorter time to clinical improvement, while patients that received Remdesivir later than 10 days post-symptom onset had a numerically higher 28 day mortality; neither finding reached statistical significance [149]. Another study found Remdesivir might provide some benefit, as treatment of COVID-19 hospitalized patients with Remdesivir shortened time to recovery. The benefit was also found to be greater if given within 10 days of symptom onset, but there was little benefit for patients already receiving mechanical ventilation or extracorporeal membrane oxygenation [141]. Taken together, these data indicate that once severe disease is established, it may be too late to use antivirals and therapies that target the aberrant host response may be more useful. In support of this hypothesis, convalescent plasma containing SARS-CoV-2 antibodies has been shown to have no effect on clinical status or mortality in critically ill patients [150]. However, therapeutics blocking the virus may be beneficial to prevent the progression to severe disease if administered early enough. Administration of monoclonal antibodies seems promising; preliminary results show that treatment with Bamlanivimab, a monoclonal antibody directed against SARS-CoV-2 spike protein, within three days of testing positive for SARS-CoV-2 reduced emergency room visits and hospital admissions in patients at high risk of disease progression [151]. Similar findings have been reported for Casirivimab/Imdevimab, two other co-administered monoclonal antibodies. All three have now been granted an emergency use authorization by the Food and Drug Administration of the United States for treatment of patients with mild to moderate COVID-19 symptoms.

### 8.2. Improving the Host Response in Severe Influenza and COVID-19: Targeting Endothelial Activation and Inflammation

Given the diminished or absolute lack of benefit to antiviral therapy in severely ill patients, potential host-targeting therapies are also being investigated. Clinical reports led to the rapid identification of host factors that were altered in severe cases of COVID-19. Among them was IL-6, a proinflammatory cytokine, which (as discussed earlier) was associated with disease progression and poor outcomes. As such, tocilizumab, an IL-6 receptor-targeting antibody, was proposed to treat the cytokine release syndrome. However, data from clinical trials has been contradictory. While cohort studies provide evidence of reduced mortality, these findings are not supported by randomized clinical trials [152,153,154]. Indeed, a recent randomized, double blind, placebo controlled clinical trial reported that treatment with tocilizumab was unable to prevent disease progression, intubation, and death, and did not decrease time to discontinuation of supplemental oxygen [155]. Conversely, tocilizumab and sarilumab, another IL-6 receptor agonist, have been found to increase the number organ support-free days to 10 and 11 days respectively, compared to 0 days for the standard of care in critically ill COVID-19 patients. They also both reduced hospital mortality to 28% (tocilizumab) and 22.2% (sarilumab) compared to 35.8% in the control group [156].

Efforts are currently underway to identify other potential therapeutic targets for the treatment of COVID-19. Multiple studies have done genome-wide CRISPR screens in an attempt to uncover host factors that are important for coronavirus infection and replication. A major limitation of these studies is that the authors of these studies only use epithelial cell lines, thus missing potential endothelial specific factors involved in pathogenesis [157,158,159,160].

Currently, the glucocorticoid dexamethasone remains the recommended treatment for hospitalized patients with COVID-19. Administration of dexamethasone reduced the incidence of death of COVID-19 patients requiring mechanical ventilation or supplemental oxygen compared to the usual care group. In addition, it shortened hospital stay and reduced the risk of progression to mechanical ventilation in patients receiving oxygen. However, dexamethasone provided no benefit in patients that did not require respiratory assistance, nor was it beneficial in patients with recent symptom onset. Interestingly, the reduction in mortality was greatest in patients with longer duration of symptoms. Taken together, these findings suggest that the beneficial effects of dexamethasone are likely due to its modulation of the host response [161]. It is possible that dexamethasone exerts its beneficial effects by acting on endothelial cells and reducing endothelial activation, proinflammatory cytokine production, and leukocyte recruitment. Glucocorticoids such as dexamethasone are well known to have anti-inflammatory effects. This may be achieved by many mechanisms such as by preventing NF-kB nuclear translocation or by preventing AP-1 binding to DNA. This results in a reduction of proinflammatory cytokine production. Treatment of cultured endothelial cells with glucocorticoids has been shown to inhibit production of IL-6, IL-8, and CCL-2 (MCP-1) among others, in addition to reducing expression of adhesion molecules such as ICAM-1, VCAM-1, and E-selectin [162]. It is also possible that dexamethasone reduces endothelial permeability, as it has been shown that treatment of endothelial cells with steroids such as dexamethasone increases junctional localization of VE-cadherin, induces VE-cadherin, occludin, and ZO-1 expression, and decreases endothelial permeability in vitro [163,164].

Interestingly, while severe disease in influenza patients is also thought to be due to the aberrant host response, treatment with corticosteroids such as dexamethasone is not recommended. In animal studies, treatment of H5N1 infected mice with dexamethasone did not prevent influenza-induced weight loss, nor did it improve hypoxemia, lung injury, or survival [165]. Furthermore, treatment with glucocorticosteroids may even be detrimental during severe influenza infection as they increase viral loads in the nasal tissue and the lungs as well as cause more severe disease [166]. In addition, treatment of patients with steroids has been associated with higher hospital mortality [167,168]. Other studies have found no effect of corticosteroids on mortality or the duration of stay in the intensive care unit, but increased mortality when corticosteroids are administered within 72 h of mechanical ventilation. Additionally, treated groups were found to have increased incidence of hospital-acquired infections [169]. Other studies have found that low to moderate doses of corticosteroids could reduce mortality in severe cases of influenza but increase mortality in mild cases [170]. It remains unclear why corticosteroid treatment appears to be detrimental in the case of influenza, but beneficial for severe COVID-19 cases. One possibility is the higher incidence of bacterial superinfection after influenza, which does not appear to be as great an issue in COVID-19 [171,172].

Other host modulating compounds have shown promise in pre-clinical models of severe infections with influenza. As mentioned earlier, endothelial-targeting compounds such as the Tie2-agonist Vasculotide and the S1P_1_ receptor agonist CYM-5442 have improved survival in mice. Both compounds decreased mortality without having any effect on viral replication, highlighting the feasibility of a host response-focused approach to treatment [65,84]. Many other host-modulating compounds targeting different components of the host response are being tested in pre-clinical models [22,137,173,174]. However, compared to COVID-19 there is far less clinical data from human patients.

### 8.3. Improving the Host Response via Anticoagulation

Various forms of anticoagulation have been proposed for the treatment of both severe influenza and COVID-19. As mentioned previously in this review, platelet inhibition is a promising treatment in pre-clinical models of severe influenza. Massive infiltration of activated platelets, platelet-endothelial adhesion, fibronectin deposition, widespread microvascular thrombosis, neutrophil-platelet aggregates and NETosis have been reported to be present in the lungs of mice infected with a lethal dose of influenza [31,105,175,176]. Importantly, the inhibition of platelet aggregation via administration of acetylsalicyclic acid to mice was shown to reduce platelet-endothelial interactions and improve survival of infected mice [31]. Prevention of platelet aggregation using other compounds has also been shown to improve survival in mouse models of severe influenza and is associated with reduced inflammation, reduction in proinflammatory cytokine levels (IL-1β, IL-6, TNF-α, and MIP-2) and reduced platelet activation [177]. Consistent with these findings, others have reported that activation of a platelet activating receptor (protease activated receptor 4, PAR4) exacerbates lung injury and worsens outcomes in influenza infected mice, while mice deficient in platelet glycoprotein 3a or treated with an antagonist of this essential platelet activating receptor displayed improved survival [105]. It has also been proposed that platelet inhibition may be beneficial during influenza infections by reducing platelet-neutrophil interactions and formation of NETs. Administration of Clopidogrel, an inhibitor of platelet activation, in combination with oseltamivir, an influenza antiviral, was shown to reduce neutrophil infiltration, neutrophil platelet aggregation, NET release, and inflammatory cytokine levels, thereby reducing lung pathology and improving survival [175]. Concurrently, it has been shown that histones, a major component of NETs, released upon influenza infection bind to platelets within thrombi and exacerbate lung pathology. This was reduced by administration of an anti-histone antibody. Blockade of extracellular histones using the anti-histone antibody also improved survival of mice when administered in conjunction with oseltamivir [176]. While pre-clinical models of influenza provide a great amount of evidence supporting the use of anti-platelet aggregation and activation therapies, their ability to protect humans has yet to be investigated in proper clinical trials. Inhibition of platelets using acetylsalicylic acid (Aspirin) in patients at risk for ARDS had no effect, decreasing enthusiasm for this strategy even though the study was a preventative rather than a therapeutic trial and was not focused on influenza infection [178].

In contrast to influenza, there is still relatively little pre-clinical in vivo investigation of anticoagulation for the treatment of COVID-19. However, given the numerous case reports of coagulation abnormalities in COVID-19 and the urgent need for therapeutics, there are ongoing clinical trials to determine if anticoagulation may be an effective treatment strategy. For instance, Heparin, an anticoagulant that acts by inhibiting thrombin and factor Xa, has been suggested as a therapeutic for COVID-19. A retrospective analysis of data from patients with COVID-19 receiving heparin or not found no difference in 28-day mortality between the two groups. However, heparin significantly reduced mortality in patients with an elevated sepsis-induced coagulopathy score (40.0% mortality compared to 64.2%) or with elevated D-Dimer levels (32.8% vs. 52.4%) [179]. It has also been reported that anticoagulation reduced in-hospital mortality among patients requiring mechanical ventilation (29.1% with median survival of 21 days compared to 62.7% with median survival of 9 days) in a large non-randomized cohort of 2773 hospitalized patients in New York city. However, this increased the risk of serious bleeding events in patients [180]. A small randomized open label phase 2 clinical trial administered Enoxaparin, a low molecular weight heparin anticoagulant that acts by forming a complex with antithrombin 3 and irreversibly inactivating factor Xa. Patients receiving therapeutic Enoxaparin had an increased ratio of partial pressure of arterial oxygen to fraction of inspired oxygen (PaO_2_/FiO_2_) 7 and 14 days after randomization compared to the standard anticoagulant thromboprophylaxis group as well as more ventilator-free days and increased ratio of successful liberation from mechanical ventilation [181]. There are ongoing clinical trials for heparin (ACTIV-4 trial, NCT04505774) and other anticoagulants such as Enoxaparin, Fondapariniux, and Argatroban (IMPACT trial, NCT04406389) to determine if they are suitable for treatment of COVID-19. However as of December 2020, three clinical trials, including ACTIV-4, have paused enrollment for critically ill patients as treatment with anticoagulants did not reduce the need for organ support. Enrollment of moderately ill hospitalized patients is ongoing [182].

## 9. Unanswered Questions

Many important questions require further investigation. For instance, it remains unclear why corticosteroids are effective for the treatment of COVID-19 but potentially worsen outcomes during severe influenza. It is possible that this is due to increased risk of secondary bacterial superinfection upon corticosteroid treatment. Secondary bacterial infections and bacterial pneumonia are common in severe influenza but seem to be less common in COVID-19 (Table 2) [35,171,172]. These secondary infections are thought to be an important contributor to influenza related mortality, and many studies have found further increased secondary infections among corticosteroid-treated influenza patients [170,183,184]. Furthermore, it has been shown in a mouse model that influenza infection itself induces a rise in serum glucocorticoids, which suppresses the antibacterial innate immune response to systemic secondary bacterial infection [185]. Elucidating the mechanisms of corticosteroid mediated protection in COVID-19 would greatly improve our understanding of the disease and its differences with other respiratory viruses such as influenza. It is also important to determine if more specific targeting of the host response, such as the blockade of specific cytokines or signaling elements, improve outcomes beyond what is seen with steroids in COVID-19. As discussed in this review, blockade of IL-6 does not seem to improve mortality in clinical trials and may even worsen the risk of nosocomial infection [155,186].

Influenza and SARS-CoV-2 have both been shown to have extrapulmonary effects in addition to their respiratory and hematologic effects described in this review. Both viruses have been reported to be associated with cardiac, hepatic, neurologic, endocrine, renal, gastrointestinal, and endocrinological effects. Influenza is associated with many neurologic effects such as influenza-associated encephalitis, post-influenza encephalitis, and Guillain Barre syndrome (GBS), while SARS-CoV-2 has also been linked to neurologic effects including GBS and encephalopathy [187,188]. However, whether extrapulmonary effects are directly due to viral infection or widespread immune dysregulation and inflammation remains unclear for both viruses. This remains an important question as it may provide guidance for the development of optimal therapies.

Finally, it is essential to determine the contribution of endothelial barrier dysfunction to pathology in COVID-19. Plasma of COVID-19 patients has been shown to induce endothelial permeability and loss of barrier integrity, contributing to severe disease [99]. This would suggest enhancing barrier integrity using a compound such as Vasculotide or Adrenomedullin may be beneficial for the treatment of COVID-19 [84,85]. However, it is unclear if enhancing endothelial barrier integrity alone would be sufficient to reduce edema and improve lung function and survival, particularly in the setting of bona fide endothelial infection; adjunctive antiviral therapy may be required. Further work is also required to tease apart the involvement of thrombosis, neutrophils, and endothelial activation to the pathogenesis of SARS-CoV-2.

## 10. Conclusions

Despite their important differences, a clear commonality that emerges from the study of severe infections with influenza and Sars-CoV-2 is the importance of the host response in determining survival. In both infections, modulation of the host response appears to be a useful strategy for improving the outcome without the risk of promoting antimicrobial resistance. Furthermore, the remarkable body of literature to come out of the last 12 months of the COVID-19 pandemic highlights the importance of the lung endothelium to acute lung injury. This critical tissue, often neglected in the study of pathogen-induced lung injury, should now be the focus of potential therapies [85].

Finally, given the importance of the lung endothelium and an aberrant immune response in both infections, it is possible that agents that are effective against virus-induced lung injury will demonstrate a broad utility against lung injury from other causes, whether due to respiratory pathogens (i.e., the next pandemic) or even extrapulmonary insults [84].

## Figures and Tables

**Table 1:** Abbreviations frequently used in this article.

Abbreviation	Full Name
ACE2	Angiotensin converting enzyme 2
ANG	Angiopoietin
ARDS	Acute respiratory distress syndrome
COVID-19	Coronavirus disease
IAV	Influenza A virus
ICAM-1	Intercellular adhesion molecule 1
IL	Interleukin
MERS-CoV	Middle Eastern respiratory syndrome coronavirus
NET	Neutrophil extracellular traps
NLRP3	NLR family pyrin domain containing 3
SARS-CoV	Severe acute respiratory syndrome coronavirus
SARS-CoV-2	Severe acute respiratory syndrome coronavirus 2
TMPRSS2	Transmembrane protease serine 2
VCAM-1	Vascular cell adhesion molecule 1
VE-cadherin	Vascular endothelial cadherin
VEGF	Vascular endothelial growth factor
vWF	von Willebrand Factor
ZO-1	Zonula occludens 1

**Table 2:** Summary of key comparisons between influenza and SARS-CoV-2.

	Influenza	SARS-CoV-2
1. Receptor	Sialic acid	ACE2/TMPRSS2
2. Infects lung endothelial cells?	Seasonal influenza (H1N1, H3N2): weak evidence Avian influenza (H5N1): Yes	Yes
3. Complications by bacterial superinfection	Frequent	Not frequent
4. Role for steroids?	Unclear, potentially harmful	Beneficial in severe cases
5a. Other host modulating drugs-barrier integrity	Vasculotide * Slit2N * CYM-5442 *	N/A
5b. Other host modulating drugs-blockade of cytokines	N/A	IL-6 receptor antagonists (tocilizumab, sarilumab)

* Only in pre-clinical models.

## Data Availability

No new data were created or analyzed in this study. Data sharing is not applicable to this article.
